# Effects of a conservative in-patient voice treatment on the voice-related self-concept

**DOI:** 10.1007/s00405-021-07021-y

**Published:** 2021-08-10

**Authors:** Bernhard Lehnert, Manfred Nusseck, Fei Lu, Annerose Keilmann

**Affiliations:** 1 Dept. Phoniatrics and Pedaudiology, ENT Clinic, University Medicine Greifswald, 17475 Greifswald, Germany; 2 Freiburg Institute for Musicians’ Medicine, Medical Faculty, University of Music Freiburg, Albert-Ludwigs-University, Freiburg, Germany; 3 Voice Care Centre Bad Rappenau, Bad Rappenau, Germany; 4 Dept. Phoniatrics and Pedaudiology, ENT Clinic, University Medical Centre Mannheim, Mannheim, Germany

**Keywords:** Voice, Dysphonia, Speech, Voice-Related Self-Concept, Rehabilitation goals, FESS

## Abstract

**Purpose:**

Observational study to determine if the voice-related self-concept as measured via the Fragebogen zur Erfassung des Stimmlichen Selbstkonzepts FESS (questionnaire for the assessment of the voice self-concept) can be improved through in-patient voice therapy.

**Methods:**

234 female and 80 male patients that underwent an intensive 3- to 4-week in-patient voice treatment due to varying types of dysphonia. After imputation of missing items but not missing questionnaires, 255 patients were eligible for FESS evaluation, 313 for VHI-12 evaluation.

The German questionnaire for the assessment of the voice self-concept (FESS) and the German 12-item short-form of the Voice Handicap Index (VHI-12) were administered at the beginning and at the end of the hospital stay. Before–after comparisons are made visually and via t test.

**Results:**

The Voice Handicap was significantly reduced, demonstrating the effectiveness of the administered therapy. Of the three scales of the FESS, the relationship with one's own voice and the awareness of the use of one's own voice was increased and thus improved. The connection between voice and emotional changes decreased significantly but only slightly.

**Conclusion:**

Conservative voice rehabilitation can not only reduce the voice handicap, but also improve the voice self-concept and the results can be measured.

## Introduction

Voice functioning plays a fundamental role in everyday life. The voice is a main source of information in social communication and it conveys not only the content of the words but also characteristic information about the speaker, such as age, gender, and emotionality [1]. Voice problems, such as hoarseness or roughness, can reduce intelligibility of interpersonal communication by impairing understanding of contextual cues [2]. Therefore, maintaining a healthy voice is also of occupational importance.

Several studies on voice therapy and treatment showed that the voice quality measurably increased after voice training [3, 4].

The majority of previous studies on the effects of voice treatments focused on the examination and changes of voice symptoms. Less studies have focused on the effects of a voice treatment on the personal relation to one’s own voice, i.e., the voice self-concept.

### Voice-related self-concept

The term self-concept describes a collection of beliefs we hold about ourselves. Self-concept is a cognitive or descriptive component of one's self, of “who we are”. Self-concept consists of many self-schemas which reflect countless aspects, such as personality, skills, abilities, occupation, hobbies, physical characteristics, gender, etc. [5, 6].

Nusseck et al. 2015 developed a questionnaire to investigate the voice-related self-concept in German language [7]. The items form three scales: Scale 1 reflects the relationship with one's own voice; scale 2, the awareness of the use of one's own voice; and scale 3, the perception of the connection between voice and emotional changes. Some publications have used sum scores, some mean scores. Substantially there is no difference. Mean scores are used within this article.

Among other researches, Nusseck et al. 2019 [8] demonstrated that an intensive voice training program significantly increased the awareness of voice use in 55 teachers as a long-term effect. So far, no comparable investigations have been done in a medical context of voice therapy.

### In-patient voice rehabilitation clinic

In Germany, there are specialized facilities for conservative in-patient voice treatment for patients with severe voice problems. In most patients, the treatment is paid by the German statutory pension insurance to prevent disease-related inability to work. In Bad Rappenau, in-patient rehabilitation for patients with severe voice disorders has been established since 1982. At that time, patients were mainly teachers. Soon patients with organic alterations of the vocal folds and with vocal cord paresis were admitted. The Bad Rappenau rehabilitation concept has been developed by a phoniatrician and a logopedist/sports therapist.

Presently, the therapy lasts for 3 or 4 weeks. All patients of a group arrive on the same day and are examined by a phoniatrician and a logopedist in a standardized fashion. The history is partly gathered by questionnaire before arrival and then supplemented by oral interview of the patient.

A complete voice examination including laryngostroboscopy is performed on arrival of the patient, repeated usually weekly and the day before discharge.

Depending on the number of patients, one or two groups of 6 to 10 patients start their treatment together and remain together during the complete stay. The fixed group is of most importance to the concept. All patients are given the opportunity to communicate about the shared problem of impaired communication, its influences on private and professional life and consecutive loss of activity and participation. The treatment consists of daily group sessions under the guidance of a logopedist, who in most cases is also responsible for the individual therapy, mostly two times weekly. The same group meets for sports therapy, especially physiotherapy, to optimize posture, to improve physical condition and endurance using exercise therapy (partly in the indoor pool) and medical Nordic walking. Patients with pain or restricted mobility have individual physiotherapy or ergotherapy and massage. All patients learn autogenic training for stress relaxation. Depending on the individual situation, about half of the patients receive psychological treatment, mostly cognitive behavioral therapy. The program is completed by lectures with moderated discussion about anatomy and physiology of voice, voice disorders, treatment and vocal hygiene. Another lecture communicates knowledge about healthy nutrition eventually completed by an individual diet counseling. Patients with problems at their working place (long inability to work, loud background noise, high vocal load necessitating technical devices) receive individual counseling by a social worker.

Building upon the Nusseck et al. [8] study with a voice training program for (healthy) teachers, German in-patient voice rehabilitation programs provide near optimal conditions to investigate the effects of voice rehabilitation on the voice-related self-concept in actual voice patients.

## Materials and methods

### Participants

The data of 234 female and of 80 male patients of the Stimmheilzentrum Bad Rappenau hospital were included in the study. These were consecutive patients with no specific inclusion or exclusion criteria except being admitted to the voice rehabilitation program and granting approval for the inclusion of their data into this study.

### Procedure

Of each included patient, we gathered gender, age and diagnostic group (either functional, organic or psychogenic dysphonia) and questionnaire data.

Questionnaire data were gathered at the beginning of the measure and at the dismissal three or four weeks later. The two questionnaires reported here were the Fragebogen zur Erfassung des Stimmlichen Selbstkonzepts (FEES) and a German abbreviated version of the Voice Handicap Index (VHI-12), both described below.

The study was approved by the Greifswald ethics committee.

### Measuring instruments

#### Voice handicap index

The Voice Handicap Index (VHI) is a standardized self-assessment questionnaire for assessing the subjective degree of having a voice handicap (Jacobson et al. [9]). It is frequently used in all kinds of diseased voice research. In this study, a German short version of the VHI with 12 items (VHI-12, formerly SSI) was used [10, 11]. The VHI has a positive scale with higher values indicating higher perception of having a voice handicap. Its range of possible values is 0 to 48.

#### The voice-related self-concept questionnaire

This paper’s introduction contains an explanation of what a voice-related self-concept is and that it can be measured via the standardized questionnaire for the assessment of the voice self-concept (Fragebogen zur Erfassung des stimmlichen Selbstkonzepts, FESS, Nusseck et al. [7]). The FESS consists of 17 items on a five-point scale focusing self-judgments on the participant’s own voice. The questionnaire contains three dimensions: Scale 1 addresses the “relationship with the own voice”, the Scale 2 assesses the “awareness of voice use”, such as the consciousness of how to use the voice and what the voice can provoke in others, and Scale 3 represents the connection between “voice and emotion”. Scales 1 and 2 are positive scales where higher values indicate a higher relationship and a higher awareness, respectively. For Scale 3, higher values show a stronger connection of changes in the emotional state and in the voice. People with higher values in this scale also showed higher psychological stress [7]. In contrast, very low values indicate a lack of connection between emotional and vocal changes.

#### Statistics

Missing values of the FESS items were found in the dataset before the voice treatment below 3% and in the dataset after the voice treatment below 1.5%. They were predicted using the multiple imputation method (with 5 imputations) which has been performed on the items of each FESS scale separately. The pooled values of the imputation were used to calculate the scale values.

Gender, age and diagnostic groups are reported descriptively, changes in all three scales of the voice-related self-concept and voice handicap index are reported as scatter plots. One-sample t tests with Welch correction are employed to confirm that mean differences before and after the hospital stay are significantly different from zero.

Statistics and graphics software were R version 4.0.5 [12] with the package ggplot2 (Wickham [13]). Multiple imputation was performed with SPSS 26 (SPSS Inc., Armonk, NY [14]).

The level of significance was set to *p* = 0.05.

## Results

### Sample description

Patient’s age ranged from 19 to 84 years with a mean of 53.3 years (♀53, ♂55 yrs). Voice disorders were categorized as “mostly organic” in 87 women and 61 men, as “mostly functional” in 116 women and 17 men and as “mostly psychogenic” in 31 women and 2 men.

We evaluated the FESS questionnaires of *n* = 255 patients and the VHI-12 questionnaires of *n* = 313 patients.

### Scale 1 of the FESS

**Fig. 1:**
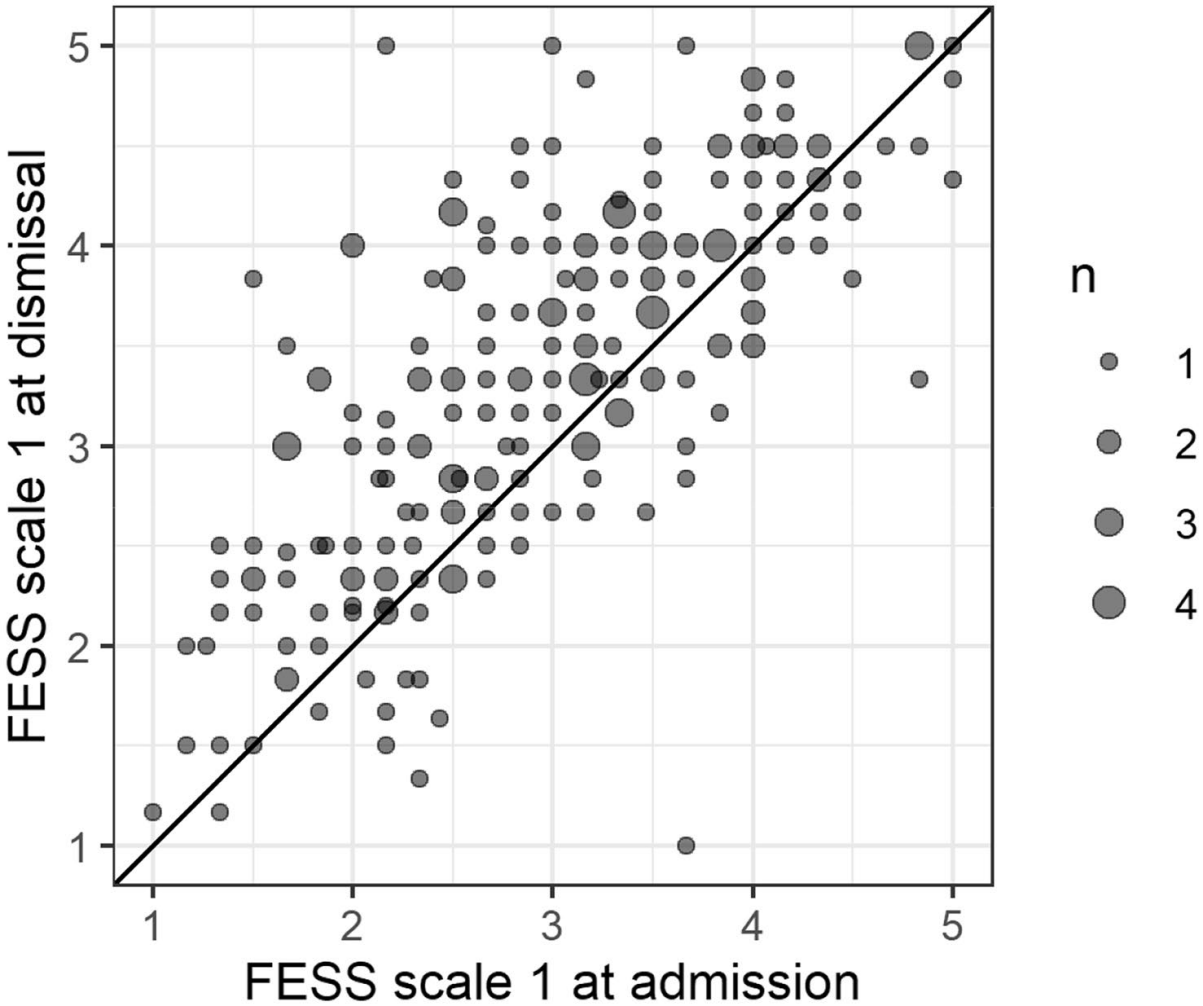
Relationship with one's own voice on admission and dismissal from hospital stay. Points on the diagonal line have identical admission and dismissal values. Values above that line reflect an increase during the hospital stay. Higher relationships with one’s own voice are considered better

Scale 1 of the FESS measures the relationship with one’s own voice. Figure 1 is a scatter plot with the scale values on admission on the x- axis and the scale values on dismissal at the y-axis.

The mean of the difference was highly significant below zero (t = -8.6812, df = 224, *p* < 0.0001), thus patients’ relationship with their own voice improved significantly during the stay (95%-CI of the mean difference: -0.48 to -0.30).

### Scale 2 of the FESS

**Fig. 2:**
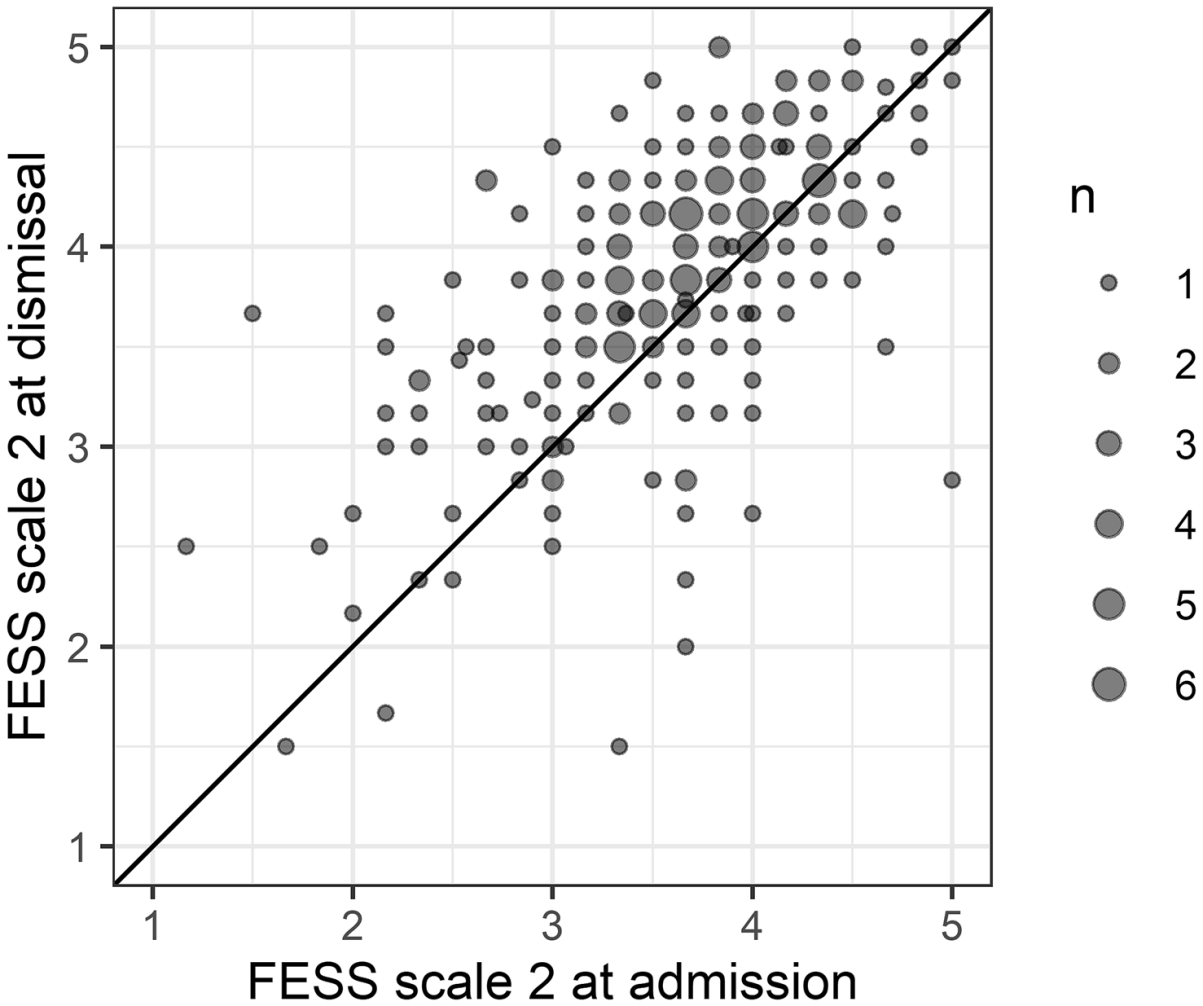
Awareness of the use of one's own voice on admission and dismissal from hospital stay. Points on the gray diagonal line have identical admission and dismissal values. Values above that line reflect an increase during the hospital stay. Higher awareness of the use of one’s own voice is considered better

Scale 2 of the FESS measures the awareness of the use of one's own voice. Figure 2 is the scatter plot of this data.

The mean of the difference was significantly below zero (t =  − 6.021, df = 224, *p* < 0.0001).

Patients’ attitude to their own voice improved significantly during the stay (95%-CI of the mean difference: -0.32 to -0.16).

### Scale 3 of the FESS

**Fig. 3:**
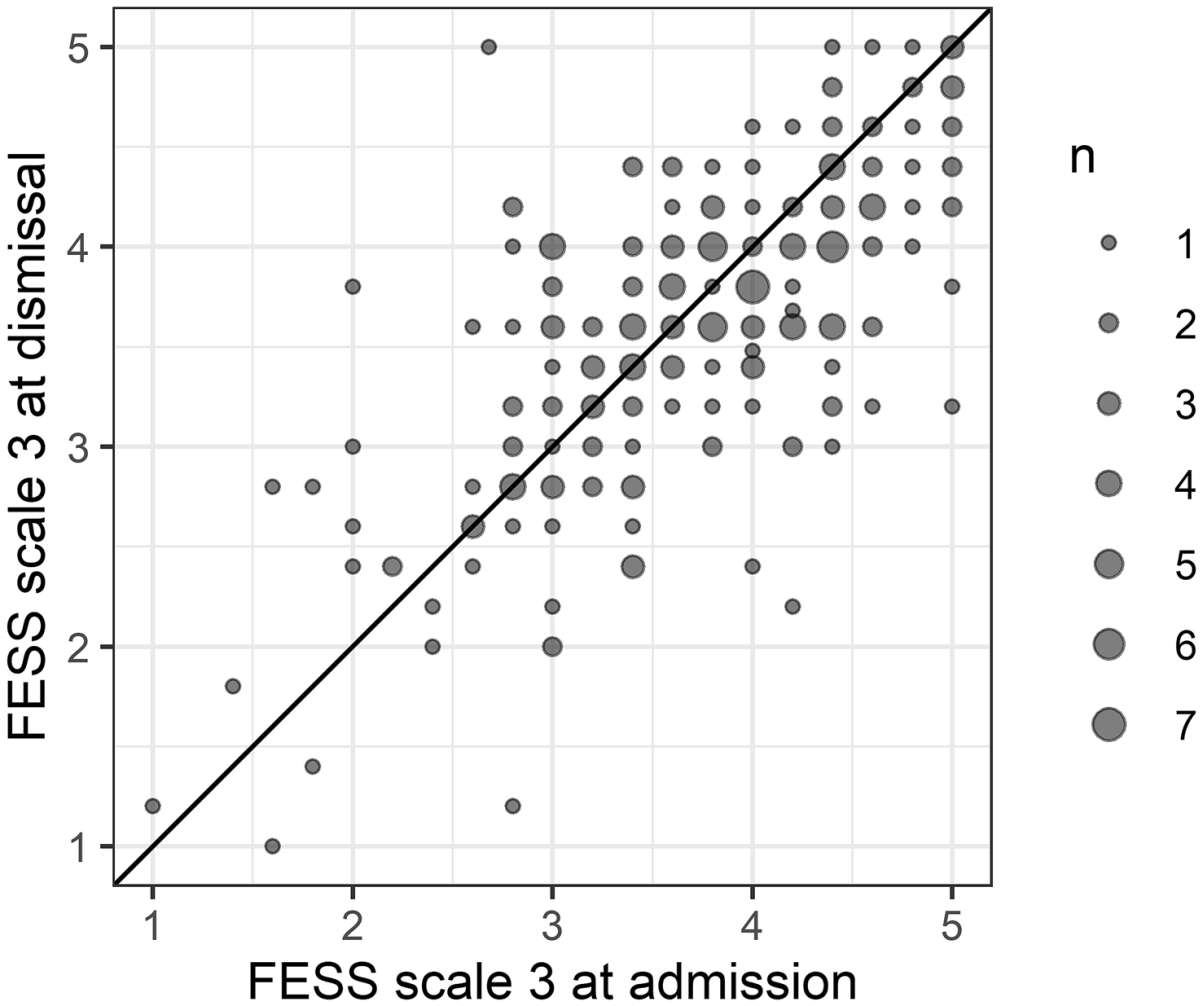
The connection between voice and emotional change was higher at admission then at dismissal

Scale 3 of the FESS measures the perception of the connection between voice and emotional changes. Figure 3 is the scatter plot.

The mean of the difference was significantly above zero (t = 2.1985, df = 224, *p* = 0.03).

Patients’ connection between voice and emotional changes decreased only slightly during the stay (95%-CI of the mean difference: 0.01 to 0.17).

### Voice handicap index

**Fig. 4:**
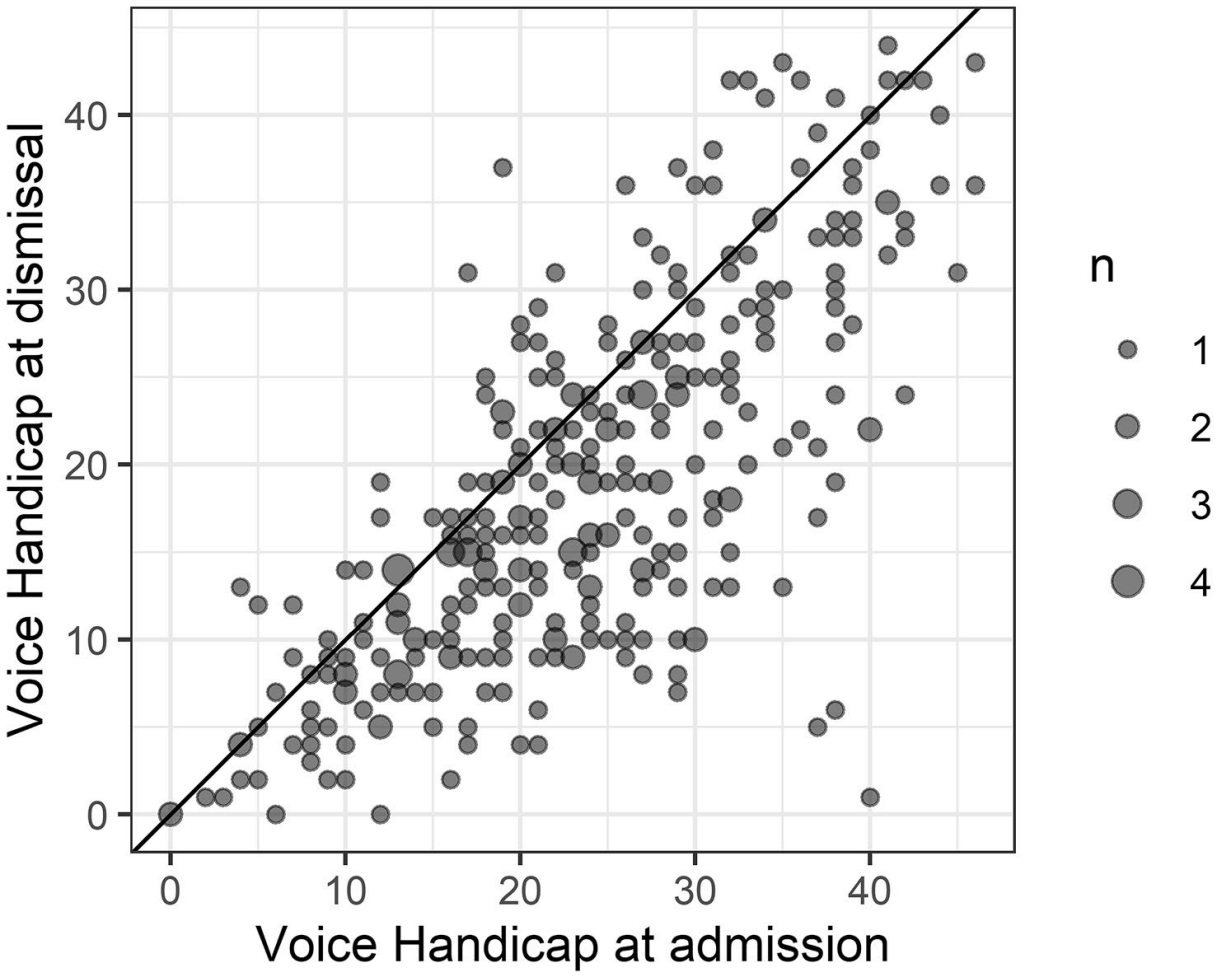
Voice handicap decreased during the stay at the Stimmheilzentrum Bad Rappenau. Lower VHI-12 values are better

The mean Voice Handicap improved from 23.5 at admission to 18.6 at dismissal (t = 11.8, df = 312, *p* < 0.0001, 95% CI of mean difference 4.1 to 5.8) (see Fig. 4).

### Post hoc analysis

**Fig. 5:**
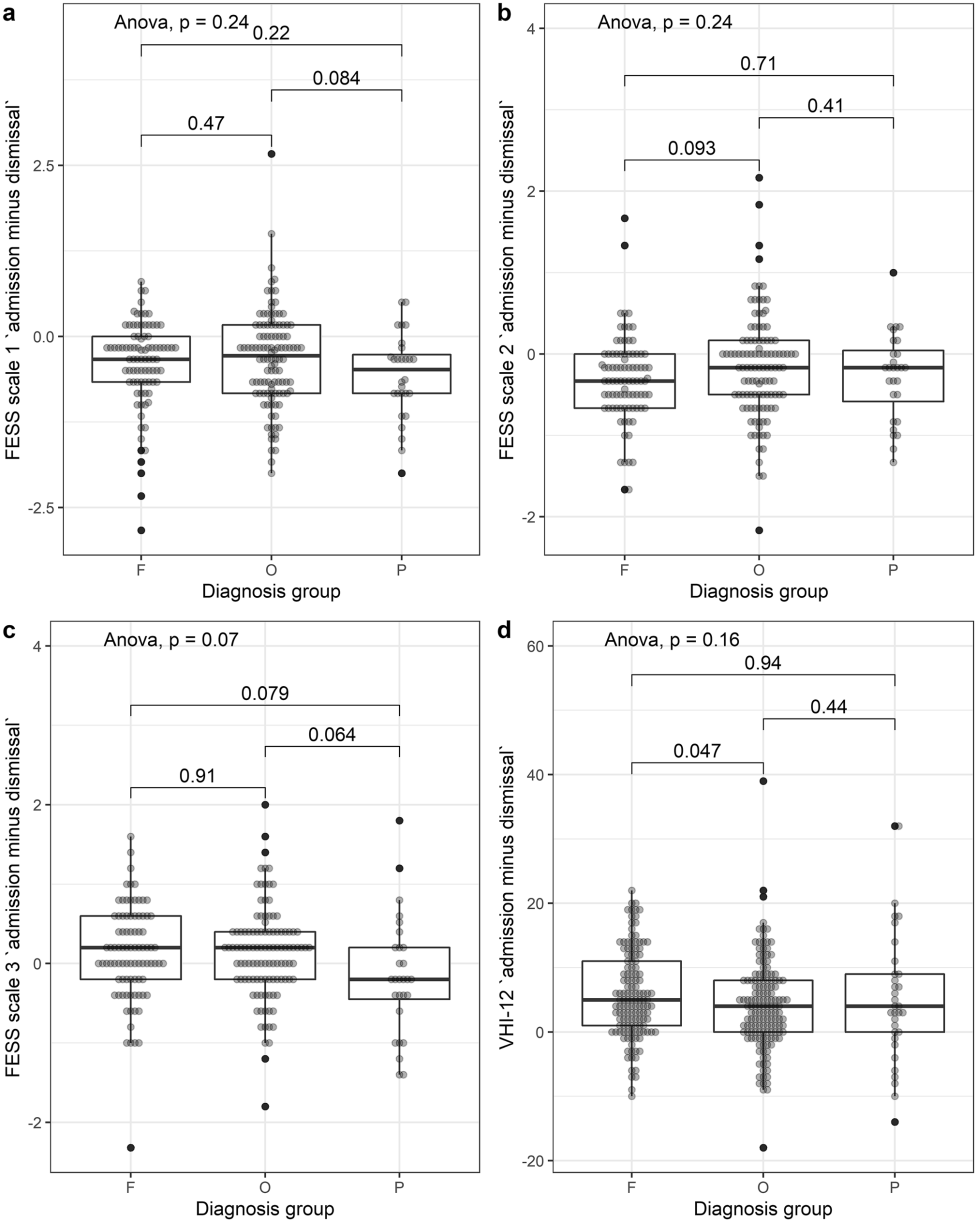
Post hoc analysis of the change in FESS scales 1 through 3 (plot a to c) and VHI-12 (plot d) by diagnosis groups (“F” mostly functional, “O” mostly organic, “P” mostly psychogenic). In plot C, we see a trend for patients with mostly psychogenic dysphonia to leave the hospital with higher scores whilst everybody else tends to leave with lower scores. Omnibus p values are from one-way ANOVA, pairwise p values from Welch-corrected t tests, no corrections for multiple testing

In a not-planned-ahead exploratory analysis, we compared how the different FESS scales and VHI-12 behaved in different diagnosis groups (O—mostly organic dysphonia, F—mostly functional dysphonia, P—mostly psychogenic dysphonia) as compiled in Fig. 5. Some of these comparisons depict trends: In Fig. 5c, depicting the FESS trait “voice and emotions”, we see a trend for patients with mostly psychogenic dysphonia to leave the hospital with higher scores whilst everybody else tends to leave with lower scores. This might be considered for further inquiries.

## Discussion

Previous studies have shown that patients’ attitude, awareness and emotional sensitivity regarding their voice differ widely. One may assume that these factors play a certain role in a patient's voice behavior and voice problem-related suffering after the therapy has been completed. Our data are proof that intensive voice therapy does not only influence voice and voice handicap but also the voice-related self-concept.

The before and after differences may seem small at first sight. However, admission to the hospital facility is granted only after an administrative act that comprises formal application. Patients will usually have had voice problems over many years, most of them will have had extensive outpatient voice therapy before the application for the in-patient treatment. So, whatever influence voice therapy has on the self-concept, much of that influence will already have influenced the before-admission measurement, the low-hanging fruits will already have been picked.

Neither the concepts of the therapy nor the implementation details of the Stimmheilzentrum Bad Rappenau were changed for the purposes of this study. Whether and/or how therapy should be oriented towards change in the voice-related self-concept was not a subject of this study, but it is a legitimate question, whether efficacy of voice therapies should be measured only by measures of voice quality and voice handicap or influence on a patient’s attitudes as well.
